# Optimization of Process Parameters for Steel Wire-Reinforced Polylactic Acid Composites Produced by Additive Manufacturing

**DOI:** 10.3390/polym17050624

**Published:** 2025-02-26

**Authors:** Turker Turkoglu, Ahmet Cagri Kilinc

**Affiliations:** 1 Department of Mechanical Engineering, Balıkesir University, Balıkesir 10145, Turkey; 2 Department of Mechanical Engineering, Osmaniye Korkut Ata University, Osmaniye 80010, Turkey; ahmetcagrikilinc@osmaniye.edu.tr

**Keywords:** additive manufacturing, composites, continuous fiber, mechanical properties, design of experiment, characterization

## Abstract

The mechanical performance of Fused Deposition Modeling (FDM)-produced polymer composites is highly dependent on processing parameters; however, most studies focus on unreinforced polymers, leaving a gap in understanding how these parameters influence continuous wire-reinforced composites. This study addresses this gap by investigating the effect of hatch spacing and layer thickness on the tensile properties of steel wire-reinforced PLA composites. The Taguchi method was employed to systematically optimize mechanical performance, using an L9 orthogonal array to evaluate tensile strength across different process conditions. The results showed that layer thickness was the most influential factor, contributing to 75.861% of the total variance (F = 60.90, *p* = 0.001), followed by hatch spacing (21.647%, F = 17.37, *p* = 0.010). The highest tensile strength of 231.61 MPa was obtained at a hatch spacing of 0.4 mm and a layer thickness of 0.2 mm, confirming the importance of optimizing these parameters to improve interfacial bonding and minimize defects. Signal-to-Noise (S/N) ratio analysis further validated these optimal conditions, with the highest S/N ratio of 47.29 observed under the same settings. This study provides a structured approach to optimizing process parameters for metal-reinforced polymer composites, contributing to the development of stronger, more reliable FDM-produced composite materials.

## 1. Introduction

Additive manufacturing technology has emerged as a significant innovation in engineering and industrial sectors. Its advantages over traditional manufacturing methods, such as design flexibility, production speed, and cost efficiency, have made this technology preferred in many application areas [1,2,3,4,5,6]. One of the most commonly used methods in additive manufacturing is Fused Deposition Modeling (FDM), which shapes objects by depositing thermoplastic materials in layers [7].

Polylactic acid (PLA) is a thermoplastic preferred for its biodegradable and environmentally friendly properties. Its low cost and ease of processing have made PLA a widely used material in various engineering and medical applications. PLA is commonly used in biomedical devices, packaging materials, and 3D printing filaments. However, the mechanical properties of PLA can be insufficient for some applications [8].

The necessity to improve the mechanical properties of PLA, such as tensile and flexural strength, is crucial for its broader application range. Continuous fiber reinforcements are widely used to enhance the mechanical strength of composite materials. Continuous fibers have significant potential to improve the strength and durability of composites. The combination of continuous fibers with different matrix materials is an effective method to optimize the performance of composite materials [9,10,11]. Continuous fiber reinforcements such as basalt fibers [12], aramid fibers [13], carbon fibers [14], glass fibers [15], and natural lignocellulosic fibers such as flax [16] are widely used to enhance the mechanical strength of composite materials. Stainless steel continuous fibers are an ideal reinforcement material for enhancing the mechanical performance of composites due to their high strength, thermal conductivity, and corrosion resistance. It is known that steel retains its strength at high temperatures and is resistant to chemical effects [17]. In this study, the use of steel continuous fibers within a PLA matrix was investigated.

Most studies in the literature focus on short fiber, particulate, or discontinuous metal reinforcements in polymer matrices, often incorporating these reinforcements by mixing them into the filament before extrusion. In contrast, this study employs continuous stainless steel wire reinforcement, which is directly embedded into the PLA matrix during the FDM process using a nozzle impregnation method. This approach enhances load transfer efficiency, interfacial bonding, and mechanical integrity, providing superior mechanical performance compared to traditional discontinuous reinforcement techniques. A review of the literature revealed that studies have primarily focused on unreinforced polymers, emphasizing the significant influence of manufacturing parameters on mechanical properties. Trindade et al. [18] evaluated the mechanical performance variations in nine different polymer-based materials, including PLA, based on print orientation. Specimens were produced in horizontal and vertical directions and subjected to tensile, flexural, and compression tests, analyzing the effect of print orientation on mechanical properties. The results indicated that mechanical performance varied with print direction and exhibited material-dependent behavior.

The influence of manufacturing parameters on mechanical performance has also been examined from different perspectives. Rivera-López et al. [19] investigated the effect of nozzle temperature on the mechanical properties of PLA specimens produced via the FDM method, revealing that insufficient melting at low temperatures or excessive material accumulation at high temperatures altered mechanical performance. The optimal printing temperature range was determined as 220–240 °C. Similarly, Vázquez-Silva et al. [20] analyzed the impact of infill density on mechanical properties, reporting that an increase in infill density enhanced mechanical strength but reduced ductility. The highest tensile, flexural, and compressive strengths were measured as 107.53 MPa, 114.32 MPa, and 63.96 MPa, respectively. Beníček et al. [21] assessed the effects of nozzle diameter, layer height, and printing temperature on PLA, PMMA, and PETG materials, demonstrating that smaller nozzle diameters and lower layer heights contributed to improved mechanical performance.

A common aspect of these studies is their focus on unreinforced polymers. While the effects of manufacturing parameters on mechanical properties have been extensively studied in the literature, limited research has explored these effects in continuous wire-reinforced polymer composites. In this context, this study provides an original contribution to the literature by investigating the impact of manufacturing parameters on the mechanical performance of steel wire-reinforced PLA composites produced via FDM.

The expected outcomes of this study include a significant improvement in the mechanical performance of PLA/stainless steel continuous wire composites by systematically optimizing process parameters. Unlike conventional polymer-based composites, the integration of continuous metal reinforcement provides superior load transfer, interfacial bonding, and mechanical reliability, which are essential for structural applications. The results demonstrate that process optimization significantly enhances tensile strength, making these composites suitable for high-performance applications requiring both lightweight design and high mechanical strength. Furthermore, the findings of this study contribute to the broader adoption of PLA-based composites by addressing one of their primary limitations—mechanical weakness. The enhanced mechanical properties achieved through optimized printing parameters expand the application potential of PLA in engineering, automotive, aerospace, and biomedical fields, where sustainable and high-strength materials are increasingly sought after. Additionally, this study promotes the use of environmentally friendly and biodegradable PLA by demonstrating its capability to be reinforced with metal wires, thereby extending its applicability to structural and load-bearing applications that traditionally rely on non-biodegradable composites. Future research may further explore multi-material hybridization, long-term durability, and functional enhancements to maximize the usability of these composites in industrial applications. The scope of this study is limited to examining the mechanical properties of composites produced by combining PLA matrix and steel continuous wires. The effects of process parameters on the performance of the composites were evaluated through systematic optimization using the Taguchi method. This approach allowed for the identification of optimal parameter settings to enhance the mechanical properties of the composites. The objective of this study is to investigate the mechanical properties of composites produced by combining a PLA matrix and steel continuous wires. The FDM method was used for production, with PLA and continuous steel wire fed into the system simultaneously to achieve additive manufacturing. Tensile tests were conducted on the produced composites to evaluate their mechanical performance.

## 2. Materials and Methods

### 2.1. Materials

Clear polylactic acid (PLA) filament (Esun Industrial Co., Ltd., Shenzhen, China) (clear refers to polymer does not contain filler, pigment, or masterbatch) with a diameter of 1.75 mm was used as matrix material for production of continuous steel wire-reinforced composites. According to the data sheets published by the seller, properties of the clear PLA filament are given in Table 1. Continuous steel wire was used as a reinforcement.

### 2.2. Three-Dimensional Printing of Continuous Wire-Reinforced Composites

**Figure 1:**
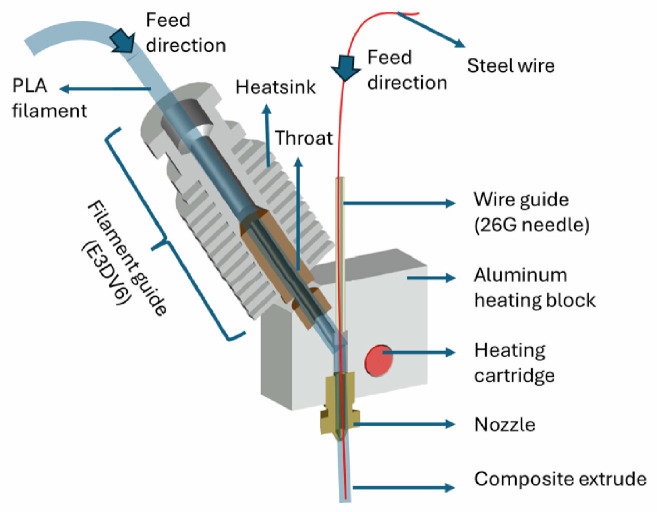
Schematic representation of hot-end system.

A hot end made of aluminum was produced to provide a linear path based on the designs of Tian et al. [23] and Ibrahim et al. [24] for 3D printing of continuous metal wire-reinforced composites by nozzle impregnation method. Th heatsink and throat parts of the commercially available hot end called E3DV6 were used as the PLA filament guide and 26-gauge stainless steel dispensing needle with an inner diameter of 0.25 mm was used as a metal wire guide. The guides were connected to the aluminum heating block. A schematic representation of the continuous wire hot-end system is shown in Figure 1.

**Figure 2:**
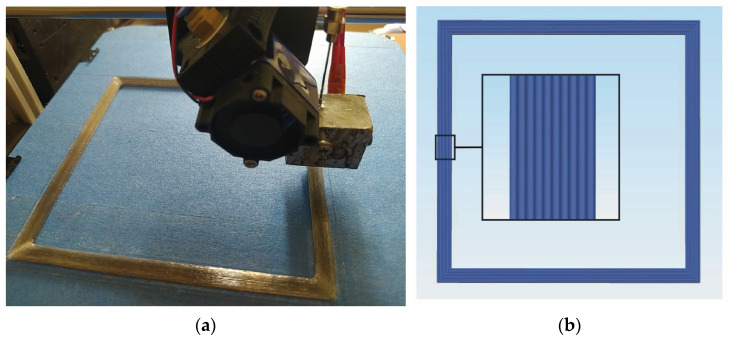
(**a**) 3D printing of frame-like part, and (**b**) representative printing path.

Custom made a cartesian-type FDM 3D printer whose printhead moves in the X and Z axis while the build plate moves in the Y axis was used for the printing process. A printing path was used for the production of composites proposed by Kuschmitz et al. [25]. Frame-like parts were printed, providing an opportunity for four test specimens to be used directly for tensile tests by cutting the edges of the frame. An image of frame-like parts and printing path are shown in Figure 2a and Figure 2b, respectively.

To minimize the nozzle wear, a hardened steel nozzle with an outlet diameter of 0.6 mm was used. Printing temperature and printing speed were fixed at 210 °C and 10 mm/s, respectively, during the 3D printing process. The printing process was performed with different thickness layers and hatch spacing parameters. Hatch spacing and layer height values were set in the range of 0.4–0.6 mm and 0.2–0.4 mm, respectively. Hatch spacing and layer height ranges were determined based on trials. Printing parameters are given in Table 2.

### 2.3. Characterization

#### 2.3.1. Scanning Electron Microscopy (SEM) Analysis

Morphological properties of both the steel wire and fracture surface of the composite specimens subjected to tensile testing were analyzed using a scanning electron microscope (Zeiss Gemini 500, ZEISS Microscopy, Oberkochen, Germany). The fracture surface of the composite specimens was Au coated using a sputter coater to achieve a ~6 nm layer.

#### 2.3.2. Thermogravimetric Analysis (TGA)

The thermal behavior of the unreinforced PLA and composites were investigated using TGA (Hitachi Exstar. Model: SII TG/DTA 7300, Tokyo, Japan). TGA was performed at a heating rate of 10 °C/min from 30 to 600 °C under a N_2_ atmosphere with a gas flow rate of 100 mL/min.

#### 2.3.3. Microstructural Examination

The 3D printed composite specimens were cut and immersed in epoxy resin for optical microscope investigation. For grinding the surfaces under water, 80 to 1200 grit SiC sandpapers were used. A Leica dm1000 optical microscope (Mannheim, Germany) was used for our investigations. Reinforcement volume fractions of the 3D printed continuous wire composites were determined from cross-sectional microstructural images by using the ImageJ (V 1.24) analyze program.

#### 2.3.4. Tensile Testing

**Figure 3:**
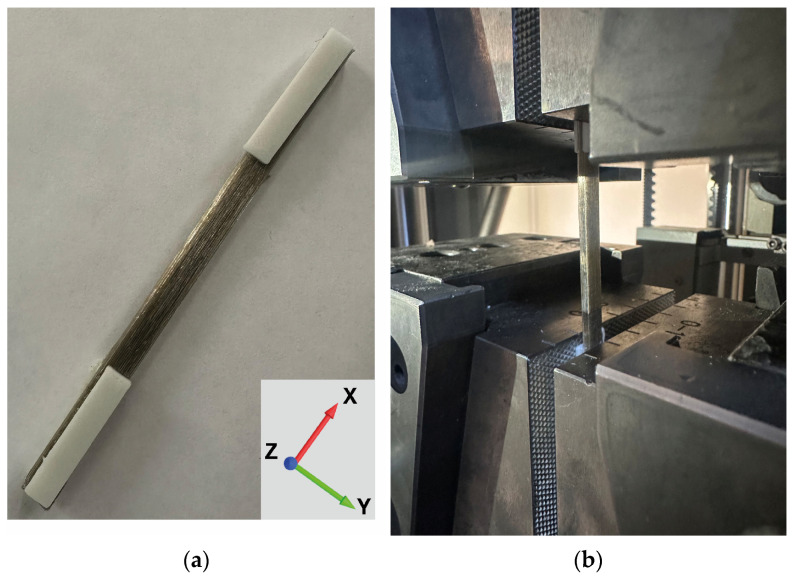
(**a**) Tensile testing specimen, and (**b**) test setup.

The dimensions of the samples were fixed as 110 mm × 6 mm × 2.4 mm (x × y × z). Tests were performed with a crosshead speed of 2 mm/min. Figure 3a and Figure 3b show the tensile testing specimen and test setup, respectively.

### 2.4. Taguchi Analysis

In this study, the optimization of the process parameters to produce steel wire-reinforced PLA composites via the Fused Deposition Modeling (FDM) method was conducted using the Taguchi method. The Taguchi method, a robust design optimization technique, was employed to systematically analyze the influence of the process parameters and enhance the mechanical performance of the produced composites. This method provides an efficient approach to optimizing manufacturing processes by minimizing variability and improving quality with a reduced number of experiments.

The larger-the-better approach was adopted in this study as the objective function, considering tensile strength as the key response variable. This approach is commonly used when the goal is to maximize a desired property, which, in this case, is the tensile strength of the composite specimens. The larger-the-better characteristic ensures that the process parameters are optimized to achieve the highest possible tensile strength while minimizing inherent variability and process-induced defects. The Signal-to-Noise (S/N) ratio for the larger-the-better approach, which is adopted in this study to maximize the tensile strength of the composites, is given in Equation (1).

(1) $$\frac{S}{N}=-10\log(\frac{1}{n}\sum_{i=1}^{n}\frac{1}{{y_{i}}^{2}})$$

where

S/N represents the Signal-to-Noise ratio,

n is the number of experimental trials,

y_i_ denotes the measured response value for each trial.

This formulation ensures that the process parameters are optimized to achieve the highest possible mechanical performance while minimizing variability.

An L9 orthogonal array was selected to evaluate the effects of key process parameters, including hatch spacing and layer thickness, on the tensile properties of the composites. Each parameter was analyzed at three different levels to systematically assess their impact on mechanical performance. The Signal-to-Noise (S/N) ratio was calculated for each experimental condition to determine the optimal parameter combination, ensuring improved interfacial bonding and mechanical integrity. The analysis was performed using statistical software (MINITAB 16.1) to identify the most significant factors influencing tensile strength and to provide insights into the contribution of each parameter to the overall performance of the composites. The results obtained from the Taguchi optimization were validated through experimental testing, demonstrating the efficacy of the adopted approach in enhancing the mechanical properties of steel wire-reinforced PLA composite. The process parameters and their respective levels considered in this study are presented in Table 3. Hatch spacing and layer thickness were selected as the primary factors influencing the mechanical properties of the steel wire-reinforced PLA composites. Each factor was evaluated at three levels: low (−1), medium (0), and high (+1), as shown in Table 3, to systematically investigate their impact on the tensile strength of the produced composites.

The experimental design used in this study follows an orthogonal array approach to systematically evaluate the effects of hatch spacing and layer thickness on the mechanical properties of the steel wire-reinforced PLA composites. The selected process parameters were investigated across nine experimental runs, as shown in Table 4, ensuring a comprehensive analysis of their interactions and individual contributions to tensile strength.

## 3. Results and Discussion

### 3.1. Thermogravimetric Analysis (TGA)

**Figure 4:**
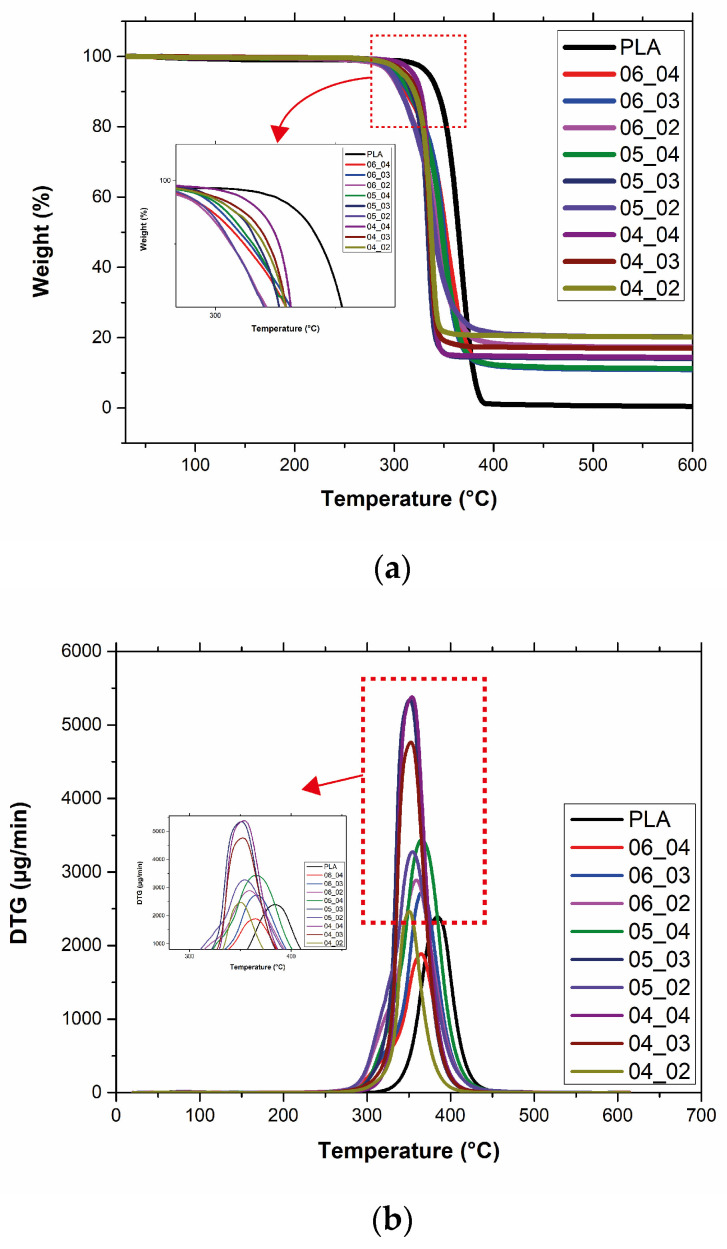
(**a**) TGA and (**b**) DTG curves of neat PLA and composites.

Thermogravimetric analysis (TGA) records the mass change in a sample depending on the user-defined heating rate under a controlled atmosphere. In this study, TGA was used to determine the thermal stability, the decomposition temperature, and the weight loss resulting from the thermal decomposition of continuous steel wire-reinforced composites 3D printed with varying hatch spacing and layer heights and neat PLA. The TGA and DTG curves of the samples are shown in Figure 4a and Figure 4b respectively. The TGA curves of the samples are shown in Figure 4a, where no significant decrease in weight value was observed up to 298.44 °C (T_onset_). At 298.44 °C the weight value of the sample decreased to 98.79 wt.% for neat PLA. At this point, decomposition of neat PLA started and after this, a sudden decrease in weight was observed. The weight decrease continued up to 384.27 °C (T_endset_) with a maximum degradation rate at 367.46 °C (T_max_). The maximum degradation rates of the composites (T_max_) were determined by using DTG curves as seen in the Figure 4b. At 367.46 °C the weight value of the sample decreased to 4.79 wt.% and after 600 °C a negligible amount of specimen remained with a weight percent of 0.41 wt.% which represents the char content of neat PLA. As depicted in Figure 4b, the DTG curves display single peaks due to the decomposition of matrix material PLA. Sztorch et al. [26] indicated that the thermal degradation of PLA is caused by random chain scission or specific chain-end scission because of its repeated aliphatic ester structure. It was observed that the degradation onset temperatures shifted to slightly lower values with the incorporation of steel wires into the PLA matrix. T_onset_, T_max_, and T_endset_ values decreased to 274.92 °C, 336.83 °C, and 369.16 °C, respectively. It is indicated in the literature that the incorporation of metallic particles shifts the decomposition temperatures to lower values by acting as degradation catalysts and increasing the thermal conductivity of the material which may play a role in the decomposition process [26,27,28]. The remained weights of specimens increased with decreasing layer height and hatch spacing values of 3D printing. Similarly, the remaining weights increased from 0.41 wt.% for neat PLA to 20.17 wt.% for 04_02 composite specimen with decreasing layer height and hatch spacing values which correspond to the reinforcement fraction by weight with a negligible amount of char residue derived from the decomposition of the PLA matrix.

### 3.2. Microstructural Characterization

**Figure 5:**
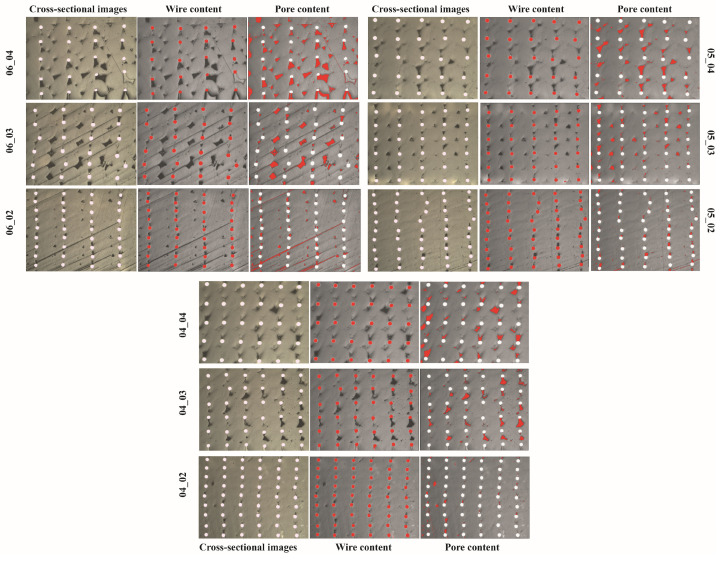
Microstructural images of composites (red color indicates wire and pore areas).

Cross-sectional optical microstructures of continuous steel wire-reinforced PLA composites 3D printed with varying layer thickness and hatch spacing values are shown in Figure 5. The bright dots observed in the figures represent metal wires, while the other parts represent the PLA matrix. The relatively light-colored areas within the matrix represent air voids. These voids are caused by the printing process and a decrease in void size is noticeable due to the decreasing layer thickness and hatch spacing parameters. The microstructure study showed that the wires within the matrix were uniformly distributed for all samples regardless of the printing parameters, as shown in Figure 5. As expected, the metal wires became closer by decreasing the layer thickness and hatch spacing values from 0.4 to 0.2 and from 0.6 to 0.4, respectively. Despite the increasing printing pressure and tracking force caused by the nozzle due to the decreasing layer thickness value [29], there was no positional change in the deposition of the wires, which were deposited uniformly on each other.

In addition, an increase in the amount of wire reinforcement per unit area was observed with decreasing layer thickness and hatch spacing values. The volume fractions and pore content calculated by the optical images based on the areal ratio are given in Table 5. The table shows that the reinforcement amount increased from 1.95 to 5.67 with decreasing layer thickness and hatch spacing values from 0.4 to 0.2 and from 0.6 to 0.4, respectively. Regardless of the printing parameters, the porosity was homogeneously distributed over the entire cross-section. With the decrease in layer thickness and hatch spacing values from 0.6 mm and 0.4 mm to 0.4 mm and 0.2 mm, respectively, a decrease was observed in the voids around the wire and between the deposited lines, and the porosity value decreased from 8.98% to 0.99%. Besides decreasing layer thickness and hatch spacing, pre-processes such as coating of continuous fibers by the matrix polymer before 3D printing, using roller supported 3D printing or compaction of 3D printed specimens using hot-pressing were effective strategies to minimize the formation of voids that occurred during 3D printing [30,31,32].

### 3.3. Tensile Testing of Composites

**Figure 6:**
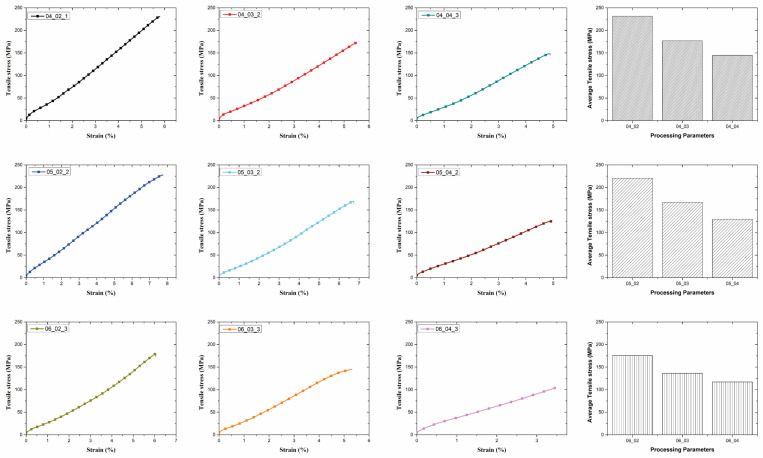
Tensile test results of composites.

The mechanical properties of the steel reinforced PLA composites produced using the FDM method were evaluated under varying hatch spacing and layer thickness parameters. The tensile strength values, calculated as the average of three samples for each parameter set, revealed significant trends associated with the production conditions (see Figure 6).

The results indicated that hatch spacing plays a critical role in determining the mechanical performance of the composites. Samples produced with a hatch spacing of 0.4 mm exhibited the highest average tensile strength (231.61 MPa) when combined with a layer thickness of 0.2 mm. This result can be attributed to the optimized overlap between the deposited layers, which enhances interlayer bonding and reduces void formation. Conversely, increasing the hatch spacing to 0.6 mm led to a notable decrease in tensile strength, with the lowest average value of 117.06 MPa recorded at a layer thickness of 0.4 mm. This reduction is likely due to insufficient overlap, resulting in weaker adhesion between adjacent lines of deposition. Thompson et al. [33] emphasized that the alignment and distribution of reinforcing elements within the matrix are critical for load transfer efficiency and overall mechanical performance, highlighting the importance of precise deposition parameters to minimize defects. Similarly, Mishra et al. [34] observed that the selection of narrower layer widths can lead to insufficient interlayer adhesion, resulting in weaker mechanical properties and reduced tensile strength. Layer thickness also significantly influenced the tensile strength of the composites. At a constant hatch spacing, reducing the layer thickness from 0.4 mm to 0.2 mm consistently improved the tensile strength. For instance, at a hatch spacing of 0.5 mm, the tensile strength increased from 129.61 MPa to 220.39 MPa as the layer thickness decreased. Thinner layers promote finer resolution and enhanced wire alignment within the matrix, contributing to improved load transfer across the composite. As Ibrahim et al. [35] highlighted, thinner layer heights allow for finer detail and improved interfacial bonding between the matrix and continuous reinforcement, further enhancing the mechanical performance of the composite. Similarly, Nguyen-Van et al. [36] observed that optimized layer heights contribute to better interfacial bonding between the matrix and reinforcement layers, effectively reducing defects and enhancing overall structural integrity. The combined effect of hatch spacing and layer thickness was evident in the mechanical performance of the composites. While both parameters individually influenced tensile strength, their interaction revealed an optimal combination for achieving superior mechanical properties. The maximum tensile strength was achieved with a hatch spacing of 0.4 mm and a layer thickness of 0.2 mm. This combination likely facilitates optimal deposition conditions, minimizing defects and maximizing the reinforcing effect of the steel wires. Previous studies have shown that voids play a significant role in influencing the tensile strength of composites. Saleh et al. [37] emphasized the importance of minimizing voids during the FDM process to enhance the mechanical properties of the produced composites. Similarly, Chacón et al. [38] highlighted that the presence of air voids (porosity) and weak bonding between fiber/matrix layers significantly affects the mechanical properties of continuous fiber-reinforced composites.

This study’s findings underscore the importance of carefully selecting processing parameters to achieve desired mechanical properties in FDM-produced composites. The improvements demonstrated in tensile strength highlight the potential of steel-reinforced PLA composites for applications requiring high mechanical performance. In light of these evaluations, a systematic approach to process optimization was undertaken using the Taguchi method, which allowed for the identification of optimal parameter settings to enhance the mechanical properties of the composites while minimizing variability. Tensile properties of various 3D printed continuous fiber composites are listed in Table 6.

### 3.4. Scanning Electron Microscope (SEM) Analysis

**Figure 7:**
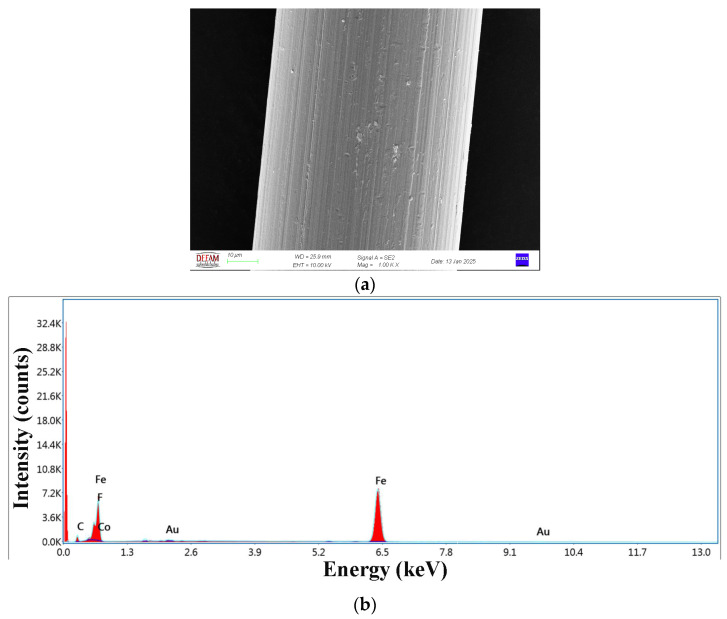
(**a**) Wire surface and (**b**) EDX graph of wire.

Surface and cross-sectional SEM images, and EDX analysis of steel wire are shown in Figure 7a,b. The SEM image (see Figure 7a) shows that the wire surface has a smooth structure. EDX analysis (see Figure 7b) proved that the wire was composed mainly of Fe and C elements. It should be noted that the presence of a high-intensity sharp peak located near 0 KeV is due to noise.

**Figure 8:**
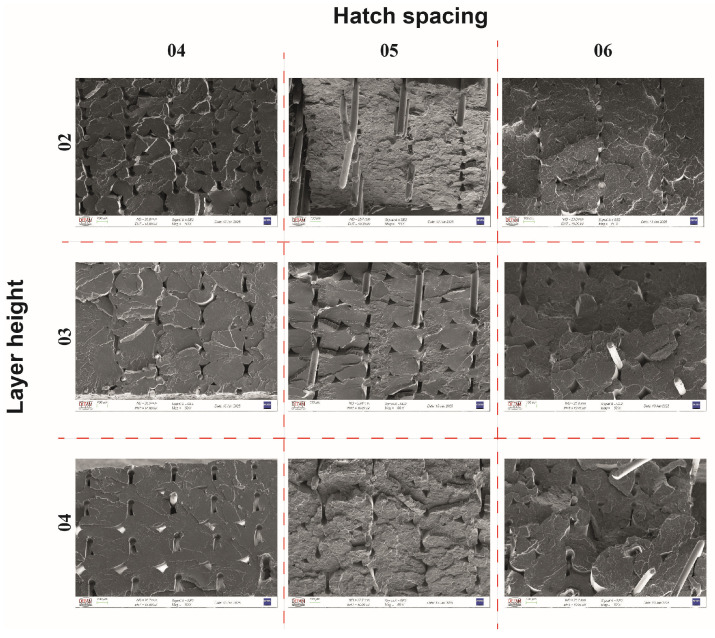
Fracture surface images of the composites.

**Figure 9:**
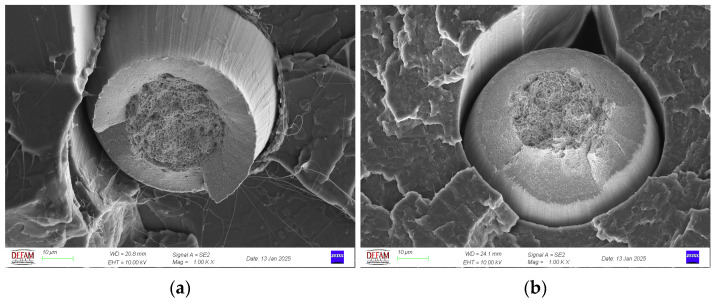
(**a**) Cup and (**b**) cone structures.

Figure 8 shows fracture surface images of continuous metal wire-reinforced composites printed with different parameters after tensile testing. The images revealed that the wires were not fully surrounded by the PLA matrix and most of the air voids were present around the wires. Similar observations were reported by other researchers [35,44], who indicated that the voids around the wires occurred due to the geometry of the extruded polymer. Mainly the wires were pulled out from the PLA matrix. Also, some embedded broken wires exist just below the fractured surface which can be attributed to elastic recovery of the steel wire (see Figure 9a,b). The cup and cone structure, which is the sign of ductile fracture the most common room temperature mechanism of failure in metals [45], is clearly seen in Figure 9a and Figure 9b, respectively.

**Figure 10:**
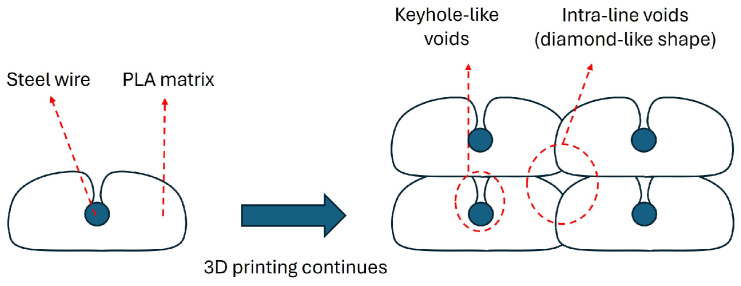
Void formation mechanism during 3D printing.

Zhang et al. [29] stated that the formation of voids in continuous fiber reinforcements is divided into two voids: inter-bead and intra-bead voids (the bead term refers to the cross-sectional shape of the individual deposited lane). Although continuous single wire was used as reinforcement in this study, a similar formation was observed. As seen in the SEM images, the voids exhibited a different profile around the steel wire and in the regions where the deposited lines contact each other. This situation is depicted schematically in Figure 10. It was assumed that during the printing process, the upper part of the wire could not be surrounded by the molten polymeric matrix which can be attributed to the high viscosity of the molten thermoplastic and therefore, it was deposited with a void (see Figure 10). As the printing process continued, it was estimated that the void on the wire was partially closed with the molten polymer by depositing a new line on this line in a similar manner and as a result, the structure took the form in Figure 10. Therefore, the void around the wire resembles a keyhole-like shape, while the void between the deposited lines resembles a diamond-like shape.

### 3.5. Taguchi Analysis Results of Tensile Test

The Signal-to-Noise (S/N) ratio values obtained from the experimental trials, presented in Table 7, provide valuable insights into the influence of process parameters on the mechanical performance of the steel wire-reinforced PLA composites. The analysis revealed that the highest S/N ratio of 47.29 was achieved in Run 1, corresponding to the combination of a hatch spacing of 0.4 mm and a layer thickness of 0.2 mm. This indicates that these processing conditions are most effective in enhancing the tensile strength, likely due to improved interlayer adhesion and optimized deposition, which minimizes defects and void formation within the composite structure.

Conversely, the lowest S/N ratio of 41.36, observed in Run 9 (hatch spacing of 0.6 mm and layer thickness of 0.4 mm), suggests that increasing both parameters simultaneously results in a suboptimal mechanical performance. This may be attributed to inadequate bonding between the adjacent layers and an increased likelihood of void formation, which adversely affects the load-bearing capacity of the composite. An overall trend observed from the data suggests that smaller hatch spacing and thinner layer thickness tend to yield higher S/N ratios, supporting the hypothesis that finer deposition enhances fiber-matrix interaction and improves mechanical properties. However, the intermediate values observed in Runs 4 and 5 indicate that a balanced approach to process parameter selection is necessary to avoid excessive processing time while maintaining mechanical integrity.

The analysis of variance (ANOVA) results presented in Table 8 provide critical insights into the significance and contribution of process parameters—hatch spacing and layer thickness—on the tensile properties of steel wire-reinforced PLA composites produced via the FDM method. The findings demonstrate that layer thickness has the most substantial influence on the tensile strength, with an F-value of 60.90 and a corresponding contribution of 75.861%, indicating its dominant role in determining the mechanical performance of the composites. The highly significant *p*-value (0.001) further supports this observation, suggesting that variations in layer thickness directly affect interlayer bonding quality, fiber-matrix interaction, and the overall structural integrity of the composite.

On the other hand, hatch spacing, with an F-value of 17.37 and a contribution of 21.647%, also plays a significant role, albeit to a lesser extent compared to layer thickness. The *p*-value of 0.010 confirms its statistical significance, emphasizing that hatch spacing influences the continuity and homogeneity of deposited layers, which in turn impacts the tensile strength by affecting interlayer adhesion and load distribution across the composite. Smaller hatch spacing is likely to enhance mechanical performance by reducing void content and promoting better bonding, while larger hatch spacing may introduce discontinuities, leading to localized stress concentrations and potential failure points. The residual error, accounting for 2.491%, suggests that the variability in tensile strength can be largely attributed to the controlled process parameters, with minimal influence from unknown or uncontrolled factors. This low residual contribution confirms the effectiveness of the experimental design and parameter selection in capturing the primary factors influencing mechanical performance.

**Figure 11:**
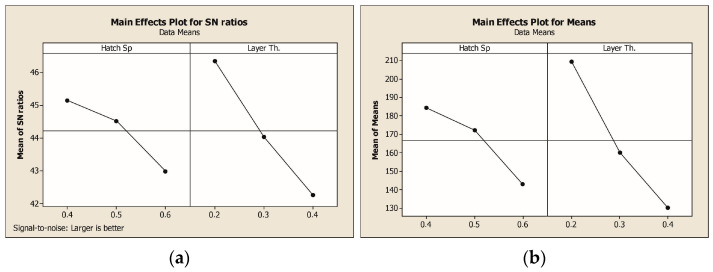
(**a**) Main effects plots for S/N ratios. (**b**) Mean tensile strength values.

**Figure 12:**
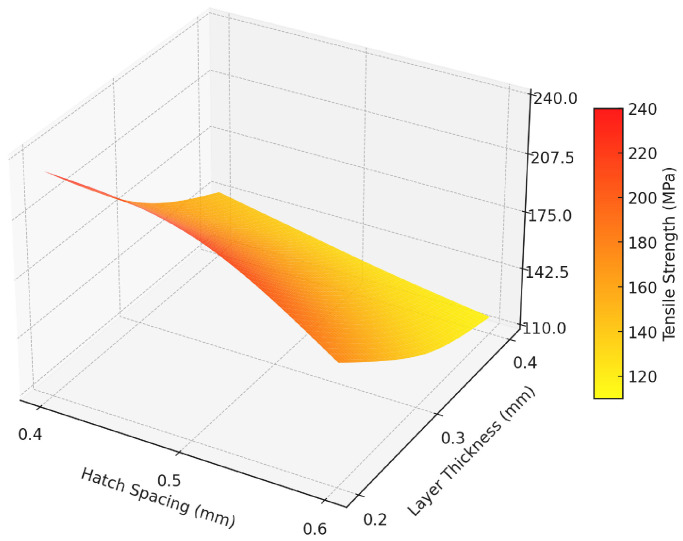
Surface plot of tensile test results.

Figure 11a and Figure 11b illustrate the main effects plots for S/N ratios and mean tensile strength values, respectively, providing valuable insights into the influence of hatch spacing and layer thickness on the mechanical performance of the composites. The plots indicate that reducing both hatch spacing and layer thickness results in higher tensile strength and improved S/N ratios, suggesting enhanced load transfer and fiber-matrix interaction at lower parameter levels. The significant decline observed with increasing layer thickness highlights its dominant effect on tensile performance, aligning with the ANOVA results. Consequently, the optimal combination for maximizing mechanical properties is identified as a hatch spacing of 0.4 mm and a layer thickness of 0.2 mm. The surface plot of tensile test results is shown in Figure 12.

## 4. Conclusions

In this study, continuous steel wire-reinforced PLA matrix composites were successfully produced. The effect of layer height and hatch spacing parameters on the mechanical and thermal properties on the composites were investigated. Layer height and hatch spacing parameters were kept between 0.4 mm–0.2 mm and 0.6 mm–0.4 mm, respectively, which were determined on the previous printing trials. The findings of this study provide valuable insights into the development of steel wire-reinforced PLA composites for advanced applications requiring enhanced mechanical properties. Unlike conventional unreinforced FDM-printed polymers, the incorporation of continuous steel wire significantly improves tensile strength and interfacial bonding, making these composites more suitable for structural and load-bearing applications. The optimization of hatch spacing and layer thickness further ensures reproducibility and reliability in manufacturing, contributing to the scalability of this approach. These results demonstrate the potential of FDM-produced metal-reinforced polymer composites in sectors such as automotive, aerospace, and biomedical engineering, where high-performance lightweight materials are required. Future research should explore additional reinforcement strategies and multi-material hybridization to further expand the application scope of these composites.

TG analysis revealed that the temperature of the beginning of decomposition (T_onset_) and the temperatures corresponding to the maximum rate of weight loss (T_max_) were shifted to the lower values by incorporation of steel wires into PLA matrix.

Microstructural characterization and SEM analysis of composites showed that the wires within the matrix were uniformly distributed for all samples regardless of the printing parameters. The cup and cone structure, which is a sign of ductile fracture, was observed for tensile tested specimens. Although some deformed fibers were observed in the structure it was concluded that the main failure mechanism for the composites was pulling out of wires from the PLA matrix. SEM analysis revealed that the composites had both void structures of keyhole-like voids and intra-line voids.

The tensile test results showed that the highest tensile strength of 231.61 MPa was achieved with a hatch spacing of 0.4 mm and a layer thickness of 0.2 mm, while the lowest tensile strength of 117.06 MPa was observed at a hatch spacing of 0.6 mm and a layer thickness of 0.4 mm. Tensile strength values decreased with increasing hatch spacing and layer thickness.

The Taguchi analysis indicated that layer thickness had the highest contribution to tensile strength with 75.861%, followed by hatch spacing with 21.647%, as shown by the ANOVA results. The highest Signal-to-Noise (S/N) ratio of 47.29 was obtained at a hatch spacing of 0.4 mm and a layer thickness of 0.2 mm, confirming the identified optimal parameters.

## Figures and Tables

**Table 1:** Properties of PLA filament [22].

Properties	Typical Value
Density	1.23 g/cm^3^
Melt Flow Index	5 (190 °C/2.16 kg)
Tensile Strength	63 MPa
Heat distortion Temperature	53 °C

**Table 2:** Three-dimensional printing parameters.

Specimen	Hatch Spacing (mm)	Layer Height (mm)	Adjacent Line Count	Layer Count
06_04	0.6	0.4	10	6
06_03	0.6	0.3	10	8
06_02	0.6	0.2	10	12
05_04	0.5	0.4	12	6
05_03	0.5	0.3	12	8
05_02	0.5	0.2	12	12
04_04	0.4	0.4	15	6
04_03	0.4	0.3	15	8
04_02	0.4	0.2	15	12

**Table 3:** The process parameters and their respective levels.

		Levels		
Factors	Units	−1	0	+1
Hatch spacing	mm	0.4	0.5	0.6
Layer thickness	mm	0.2	0.3	0.4

**Table 4:** Experimental runs.

Run	Levels	
1	0.4 mm	0.2 mm
2	0.4 mm	0.3 mm
3	0.4 mm	0.4 mm
4	0.5 mm	0.2 mm
5	0.5 mm	0.3 mm
6	0.5 mm	0.4 mm
7	0.6 mm	0.2 mm
8	0.6 mm	0.3 mm
9	0.6 mm	0.4 mm

**Table 5:** Reinforcement volume fraction of composites.

Specimen	Volume Fraction (%)	Pore Content (%)
06_04	2.02	8.98
06_03	2.50	4.19
06_02	3.71	3.13
05_04	1.95	4.55
05_03	3.53	3.16
05_02	4.83	1.6
04_04	3.35	4.56
04_03	3.74	3.12
04_02	5.67	0.99

**Table 6:** Tensile properties of various 3D printed continuous fiber reinforced composites.

Matrix Material	Fiber Material	Tensile Strength	Reference
PLA	Carbon fiber	243.53 MPa	[39]
PLA	Aramid fiber	410.25 MPa	[13]
PA	Basalt fiber	143.73 MPa	[40]
PLA	Glass fiber	241 MPa	[41]
PLA	Pineapple leaf fiber	101.51 MPa	[42]
PLA	Flax fiber	183 MPa	[43]
PLA	Copper	44.89 MPa	[44]
PLA	Nickel chromium	53.8 MPa	[44]
PLA	Steel	231.61 MPa	This work

**Table 7:** The Signal-to-Noise (S/N) ratio values.

Run	S/N Ratio
1	47.29
2	44.95
3	43.21
4	46.86
5	44.45
6	42.25
7	44.89
8	42.70
9	41.36

**Table 8:** The analysis of variance (ANOVA) results.

	F-Value	*p*-Value	Contribution (%)
Hatch spacing	17.37	0.010	21.647
Layer thickness	60.90	0.001	75.861
Residual			2.491

## Data Availability

Data are contained within the article.
